# Development of NeuroML version 2.0: greater extensibility, support for abstract neuronal models and interaction with Systems Biology languages

**DOI:** 10.1186/1471-2202-12-S1-P29

**Published:** 2011-07-18

**Authors:** Padraig Gleeson, Sharon Crook, Angus Silver, Robert Cannon

**Affiliations:** 1 Department of Neuroscience, Physiology and Pharmacology, University College London, UK; 2 School of Mathematical and Statistical Sciences, School of Life Sciences, and Center for Adaptive Neural Systems, Arizona State University, USA; 3 Textensor Limited, Edinburgh, UK

## 

NeuroML version 1.x allows specification of detailed cell and network models incorporating complex neuronal morphologies, voltage and ligand-gated ion channels, fixed and plastic synapses, and positioning and connectivity of cell populations in 3D [1]. An increasing number of freely available software packages support this version of NeuroML (http://www.neuroml.org/tool_support) and published cell and network models from the neocortex, cerebellum and hippocampus have been converted to the format. While the focus of NeuroML v1.x was on (multicompartmental) conductance based neuronal models, networks incorporating simplified neurons are widely used to investigate properties of neuronal systems [2]. These network models can be created from multiple (hierarchical) population sets with complex connectivity. At the other end of the biological scale an increasing amount of modelling work incorporates detailed subcellular signalling pathways, especially for investigating synaptic plasticity [3]. These points have been the motivation for version 2.0 of the language.

At the core of NeuroML version 2.0 is the LEMS (Low Entropy Model Specification) language. This allows Component Types to be specified which define the behaviour of Components to be used in simulations. A Component Type consists of a number of state variables and a specification of their dynamical behaviour in terms of a set of parameters. LEMS can express a wide range of dynamical models and NeuroML v2.0 will describe a core set of neuroscience specific Component Types. Current examples supported include abstract cells (e.g. I&F, Izhikevich, Adaptive Exponential I&F, FitzHugh-Nagumo), ion channels (HH, kinetic scheme based, ligand gated) and synapse models (AMPA/NMDA mediated, STP, STDP). Supporting software can handle these types natively, or support the full LEMS framework. A package for parsing LEMS is available (http://www.neuroml.org/lems), which can natively run simulations of networks of single compartment cells. This also supports export of the model in a number of formats, including NEURON, which facilitates executing multicompartmental cell models expressed in NeuroML v2.0. SBML files can also be generated from LEMS, and an import option is being developed to allow the biochemical signalling models in the BioModels database to be used with LEMS/NeuroML 2.0. An updated scheme for specifying templates for hierarchical networks has been developed and this representation, together with abstract cell models, can be exported in the emerging NineML format.

NeuroML 2.0 will encompass all functionality from v1.x, with automatic update of models possible through neuroConstruct [4] . This will make all existing detailed models available in the new version of the language. The development process for NeuroML is open to all and contributions are welcome from across the community (see http://www.neuroml.org/neuroml2). This work will facilitate the exchange of models and ideas between theoreticians, modellers and experimentalists across the neuroscience and wider systems biology communities.

This work has been funded by the NIH and the Wellcome Trust and has benefited from discussions within the INCF Multiscale Modeling Program.
